# A Novel System for the Measurement of an Evaporation Duct Using the Magnetic Coupling Principle for Power Feeding and Data Transmission

**DOI:** 10.3390/s22197376

**Published:** 2022-09-28

**Authors:** Qiang Wang, Xingfei Li, Hongyu Li, Shaobo Yang, Shizhong Yang, Linlin Ma, Jingbo Zhao

**Affiliations:** 1 College of Information and Control Engineering, Qingdao University of Technology, Qingdao 266525, China; 2 State Key Laboratory of Precision Measuring Technology and Instruments, Tianjin University, Tianjin 300072, China; 3 College of Ocean Science and Engineer, Shandong University of Science and Technology, Qingdao 266590, China

**Keywords:** gradient meteorological instrument (GMI), inductively coupled power transfer (ICPT), non-iterative air–sea flux (NAF), evaporation duct height (EDH), South China Sea (SCS)

## Abstract

Since the evaporation duct height (EDH) only covers the antenna height of most shipborne microwave radars, mastering the EDH in advance has great significance in achieving long-range target detection. In this paper, a set of hydrological and meteorological sensors based on the gradient meteorological instrument (GMI) were built to monitor the evaporation duct of the South China Sea (SCS). However, the monitoring needed to be interrupted during the battery replacement of the sensor, which could result in the loss of some important data collection. On the basis of the inductively coupled power transfer (ICPT) technology, the resonance principle was used to compensate the inductive reactance on the closed steel ring (CSR), and the energy stored in the super capacitor was introduced for data collection and return. A novel measuring system for the detection of an evaporation duct was proposed. To avoid iterative calculation by setting the initial value of the current evaporation duct models in large-scale and multi time evaporation duct prediction and diagnosis, on the basis of the non-iterative air–sea flux (NAF) model, the EDH was obtained by introducing the K theoretical flux observation method into the atmospheric refractive index equation. Finally, preliminary experimental results are presented for the detection of evaporation duct to demonstrate the feasibility and effectiveness of the proposed system. The communication accuracy rate of the proposed system was 99.7%. The system transmission power reached 22.8 W. The research results of the NAF model adaptability showed that the mean value of the EDH was 8.7 m, which was lower than the mean EDH of the SCS. The EDH calculated by the NAF model in the unstable air–sea stratification state was slightly lower than that calculated by the NPS model. The diagnosis of the EDH by the NAF model was similar to that of the NPS model, but the calculation stability of the NAF model was better.

## 1. Introduction

The interaction between the sea water and atmosphere in the South China Sea (SCS) can cause inhomogeneity in time and space of the refractive rate over the sea, which brings about the refractive error of radar measurement. A special phenomenon called evaporation duct often occurs, owing to rapid decrease in humidity, and it possesses an anomalous atmospheric refractive index structure [1,2,3,4]. Without the consideration of other influencing factors such as rainfall, the propagation of electromagnetic waves in the atmosphere is mainly determined by the structures of the refractive index, so propagation models (e.g., the terrain parabolic equation model (TPEM)) require a refractivity profile to make their calculations. It is now well established that the evaporation duct is the dominant propagation mechanism, resulting in over-the-horizon radar detection or causing a radar-blind area for microwaves at frequencies above the L-band [5,6,7,8]. Therefore, the measurement of the evaporation duct is of extreme importance because it affects most of the radar, radio communication, and navigation systems when they are operating under that condition.

Results show that the evaporation duct mainly depends on the atmospheric refractivity profile of the vertical direction. The minimum of the atmospheric refractivity responds to the evaporation duct height (EDH) [9,10]. There are many methods employed for measuring evaporation ducts. Conventionally, the evaporation duct is measured using a microwave refraction instrument [11,12], sounding balloon [13,14], or a gradient meteorological instrument (GMI) based on the tower platform [15,16], and it is predicted by the meteorological models. The refractivity from the clutter technique has recently been developed, deducing the duct height using the inversion algorithm including the genetic algorithm [17,18], the Bayesian–Markov chain Monte Carlo method (MCMC) [19,20], and the support vector machine (SVM) [21,22], among others. However, these methods have many drawbacks, such as low detecting precision, high costs, real time nature, and vulnerability to electromagnetic interference or electromagnetic exposure, which precludes them from practice in application.

Research shows that the probability of occurrence for an evaporation duct reaches 80% in the SCS due to conditions there being apt for its formation [23,24]. Thus, the height and intensity of the evaporation duct is measured by building a 0~40 m high tower platform. This platform measures the atmospheric temperature, humidity, air pressure, and other parameters at different heights and obtains the modified atmospheric refractive index profile. In general, the detection of the evaporation duct using the GMI on the basis of the tower platform has attracted individuals’ attention and is widely employed in practice. However, to obtain a high-resolution refractive index profile, it is necessary to study the evaporation duct model. Many scholars have proposed the following models based on the Monin–Obukhov similarity theory in the near-surface layer. In 1973, Jeske proposed an evaporation duct model for the propagation of the radio waves on the sea surface [25], which was later improved and revised by Paulus to form the Paulus–Jeske (PJ) model [26]. On the basis of the mesoscale forecast system, Musson-Genon et al. calculated the EDH using an analytical method and proposed the Musson-Genon–Gauthier–Bruth (MGB) model [27]. In 2000, Frederickson et al. proposed the Naval Postgraduate School (NPS) model [28]. The Non-Iterative Air–Sea Flux (NAF) model was proposed by Li et al. in 2014 and 2015 [29,30]. Although many domestic scholars have proposed many calculation methods based on the Monin–Obukhov similarity theory, due to the influence of the geographical environment factors, the adaptability of the models is not consistent. The algorithm of the PJ model is simple and fast, but it assumes that the potential refractive index, potential temperature, and potential humidity meet the Monin Obukhov similarity theory. When the actual conditions are stable, the potential refractive index does not meet the Monin–Obukhov similarity theory, so its calculation results may be wrong under the stable conditions. Moreover, its parameters are often derived from the terrestrial measurement results, so the PJ model cannot provide completely correct results. The EDH calculated by the MGB model is zero under many meteorological conditions, and the number of non-zero values will increase when the wind speed is large enough. The NPS model ignores the influence of the changes of the sea surface attachment parameters on the gradient coefficients of each parameter, and also has certain limitations. Compared with the aforementioned model, the NAF model adopts an advanced air–sea flux algorithm and extends the Monin–Obukhov similarity theory to low-wind-speed conditions. It is a relatively perfect evaporative duct prediction model in theory.

At present, to obtain the profile of the atmospheric refractive index, the GMI needs to carry a body of hydrological and meteorological sensors. Each sensor requires a cable to provide the electric energy and to transmit data though the aviation plug [31,32]. Further, when the battery of each sensor runs out and needs to be replaced, cables can let the GMI be more complicated, which can lead to high cost, short life, and hard maintenance in the high temperature and high humidity environment such as the SCS [33,34]. Inductively coupled power transfer (ICPT) technology is based on the principle of electromagnetics [35,36,37,38]. There is no direct connection between the power source and the load. On the basis of the ICPT system, the data can be transferred by using the same principle. In order to prevent the collected important data from being lost due to the interruption of monitoring during the process of replacing the sensor battery, a newly developed easy maintenance and high resistance system for measuring the evaporation duct on the basis of the magnetic coupling principle was introduced, which only required a closed steel ring (CSR) for power feeding and data transmission in the paper. In order to reduce the proportion of the reactive power and improve the overall efficiency of the non-contact power transmission system, the resonant principle was used to compensate the inductive reactance on the CSR. At the same time, the super capacitor was introduced as the energy temporary storage device of the sensor system to solve the problem of normal data acquisition after the power supply of the sensor was stopped. The built meteorological gradient meter testing equipment based on contactless power and data transmission technology was found to be suitable for the evaporation duct detection environment and was able to obtain a body of the marine hydrological and meteorological data. Since the current iterative calculation of the existing evaporative duct models requires a large number of operations, the efficiency and calculation stability of the prediction and diagnosis of the evaporative duct in a large range and multiple times was reduced. To avoid the iterative calculation by setting the initial value and to further improve the calculation stability of the existing evaporative duct models for long-term and large-scale atmospheric duct simulation prediction, the K-theory flux observation method based on the NAF model was introduced into the atmospheric refractive index equation to obtain the EDH in this paper. This paper will proceed in the following manner. First, the proposed system for detecting the evaporation duct is briefly introduced, including the structure of the proposed system and the design of inductive couplers and a sensing unit. Second, the magnetic coupling principle for power feeding and data transmission was further represented. The magnetic coupling system for power feeding and data transmission was tested. Third, according to the collected hydrological and meteorological data, the effects of the air pressure (AP), air temperature (AT), relative humidity (RH), air-sea temperature difference (ASTD), and wind speed (WS) on the EDH were analyzed by means of the NAF model. Last, the adaptability of the NAF model for predicting the evaporation duct was studied. The research results of the NAF model were compared with the NPS model. Experimental results showed that the detection of the evaporation duct was able to demonstrate the capability of the proposed system. The feasibility of the NAF model in the prediction of the EDH in the SCS was verified.

## 2. The Proposed System for Detecting the Evaporation Duct

### 2.1. The Structure of Proposed System for Detecting the Evaporation Duct

**Figure 1:**
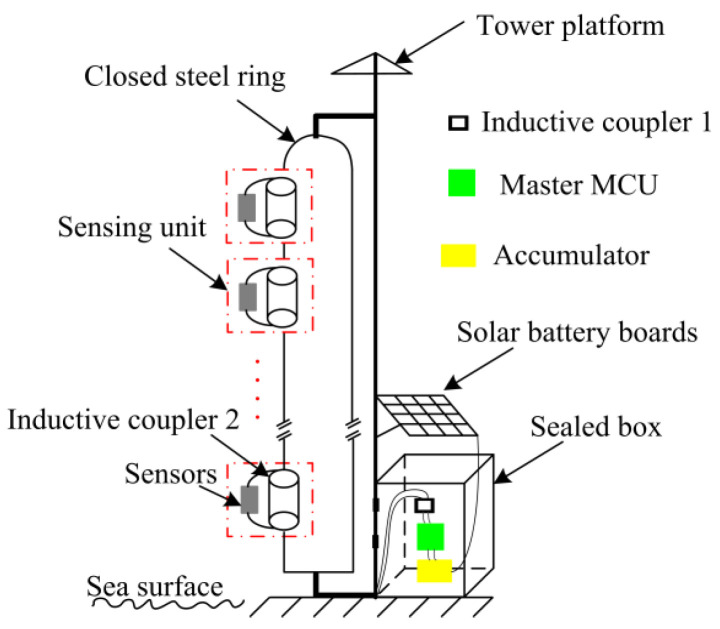
General overview of the proposed system for measuring the evaporation duct.

**Figure 2:**
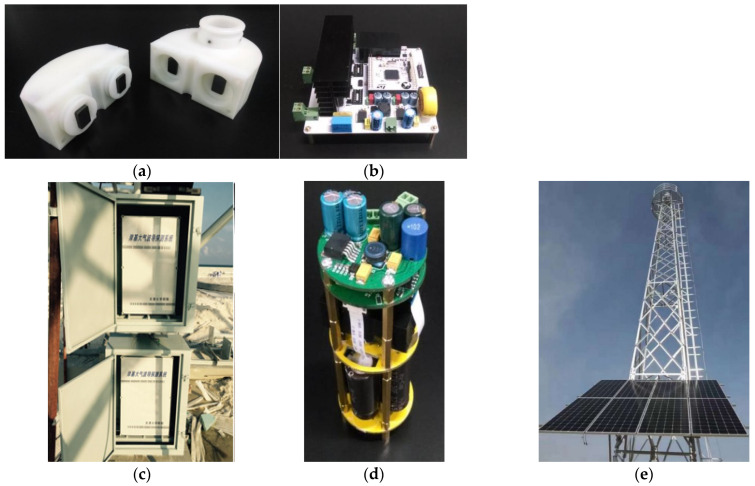
The physical photo of measurement system components of the evaporation duct. (**a**) Inductive couplers; (**b**) master circuit; (**c**) sealed box (it is used to store the solar controller); (**d**) multiple sensing units; (**e**) solar battery boards.

Figure 1 shows a general overview of the proposed system for detecting the evaporation duct, and it includes the closed steel ring (CSR), inductive couplers (IC), a multiple sensing unit, an accumulator, a sealed box, solar battery boards, and a master circuit, among others. The measurement system components of the evaporation duct are illustrated in Figure 2. The solar battery boards and accumulator provide the energy source for the experiment system. The microprocessor control unit (MCU) is the heart of the experiment system and is used to ensure the normal operation of the system. The CSR is attached to a tower platform and is equipped with five sensing units at the equidistant intervals up to 30 m height over the sea surface. The MCU, accumulator, and IC 1 are protected from the salt fog corrosion in the shielding box. According to the demand of the measuring elements, the sensing unit contains the inductive coupler 2 and circuit conversion module carrying different sensors. A half-transparent plastic mantle covers the coils in order to prevent the corrosion caused by the water and salt-fog. Inductive coupler 1 and inductive coupler 2 were installed in the same closed ring that can be thought of a core of the power-feeding transformer.

### 2.2. The Design of IC in the Experimental System

**Figure 3:**
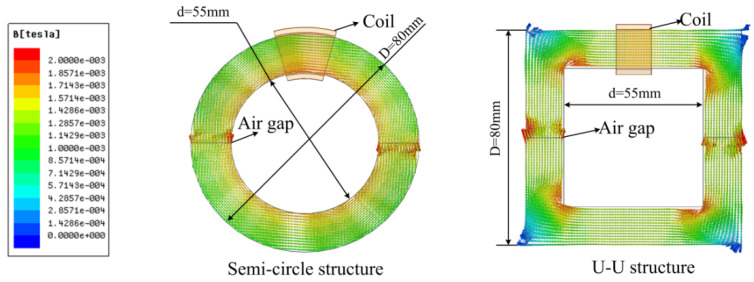
Distribution of magnetic field of the semi-circle and U-U structure core (semi-circle core: D = 80 mm, d = 55 mm, air gap = 0 mm; U-U structure core: D = 80 mm, d = 55 mm, air gap = 0 mm).

To meet the operating requirements at a high frequency, the ferrite core was used in the construction of the “core” of the IC due to its high permeability, conductivity, and saturation magnetic flux density. The CSR is required to pass though the center of the core. Therefore, a two-half buckle structure was employed in the system. In order to study the effect of the magnetic core structure on the magnetic circuit, the distribution of the magnetic field is shown in Figure 3 though Ansoft Maxwell software [35,36,37,38] when the exciting current was 0.3 A. The simulation results show that the distribution of the magnetic field of the semi-circle structure core have great advantage in uniformity and higher magnetic induction intensity in most cases compared with the U-U structure core. The coil position has less influence on the electric energy transmission when the magnetic field is in uniform distribution. On the condition of the same exciting current, the higher magnetic induction intensity can be able to produce a bigger output voltage and improve the output power. Thus, the semi-circle structure core was selected as the core of the IC in the experiment system.

**Figure 4:**
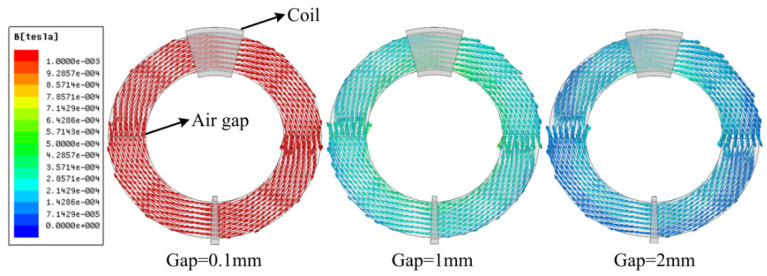
Distribution of the magnetic field of the semi-circle structure core with different air gaps (gap = 0.1 mm, 1 mm, and 2 mm, respectively).

Due to the existence of the air gap, the magnetoresistance will increase. When the number of the coil turns remains unchanged, the current in the coil will increase, resulting in the increasing copper loss of the electromagnetic coupler. The distribution of the magnetic field of the semi-circle structure core with different air gaps is shown in Figure 4. It can be seen from Figure 3 that the magnetic induction intensity in the magnetic core decreases with the air gap increasing. The air gap between the magnetic cores has a great influence on the performance of the electromagnetic coupler, and the existence of the air gap greatly reduces the equivalent magnetic permeability of the magnetic core. In order to reduce the air gap of the magnetic core, on the one hand, it is necessary to ensure the high accuracy of the magnetic core abrasive tools, especially in the levelness of the end face of the magnetic core; on the other hand, the roughness of the end face of the magnetic core is reduced, and the length of the air gap is reduced microscopically.

### 2.3. The Design of the Sensing Unit in the Experiment System

**Figure 5:**
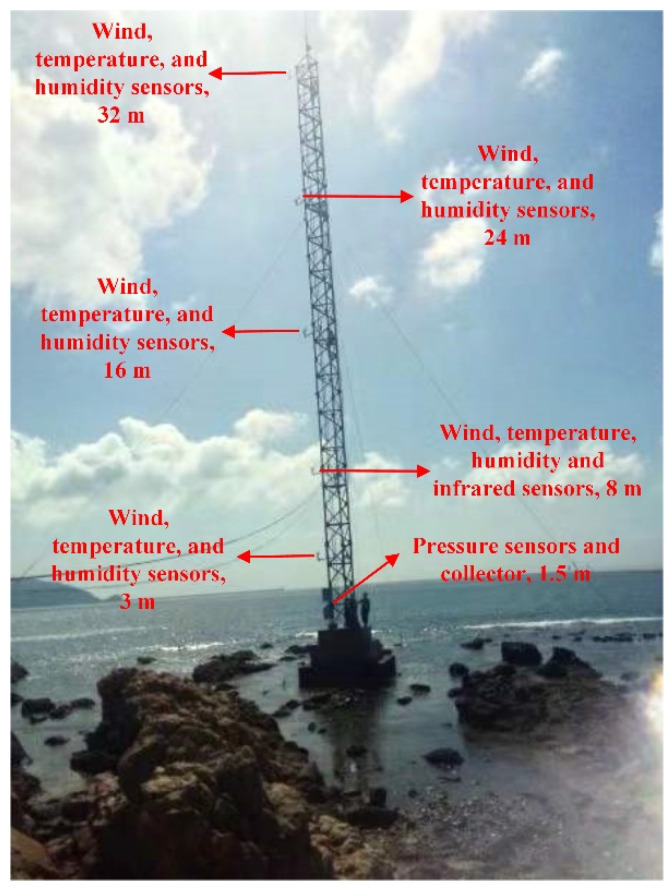
Measurement system of evaporation duct based on an iron tower platform.

In order to obtain the real time and continuous detection of the evaporation duct, an evaporation duct measurement system was designed to realize the automatic and continuous observation of the evaporation duct. The evaporation duct measurement system is generally based on an iron tower (generally higher than 40 m) as a platform, including a sensor subsystem, a data acquisition subsystem, a power supply subsystem, and a communication subsystem, as shown in Figure 5.

It is reported that the average height of the evaporation duct in the SCS is 13 m, and the EDH is generally lower than 40 m. In order to obtain the section pan of the atmosphere refractivity index, the height of sensing unit was designed at 3 m, 8 m, 16 m, 24 m, and 32 m over the ocean. Each sensing unit contained a wind sensor (RM Young, Traverse City, MI, USA), a temperature sensor, and a humidity sensor (Vaisala, Vantaa, Finland). The 8 m height of the sensing unit added an air pressure sensor (Vaisala, Finland) and an infrared sensor for measuring the surface temperature of the sea water (Apogee, Bournemouth, England). It is worth noting that the atmospheric duct is very sensitive to the hydrological and meteorological elements, so all sensors were selected according to the parameters of accuracy and precision. The data of all sensors were stored in the collector after processing, and the sampling frequency of the collector was 1 s/time. The parameters of all sensors are shown in Table 1.

## 3. The Magnetic Coupling Principle for Power Feeding and Data Transmission

**Figure 6:**
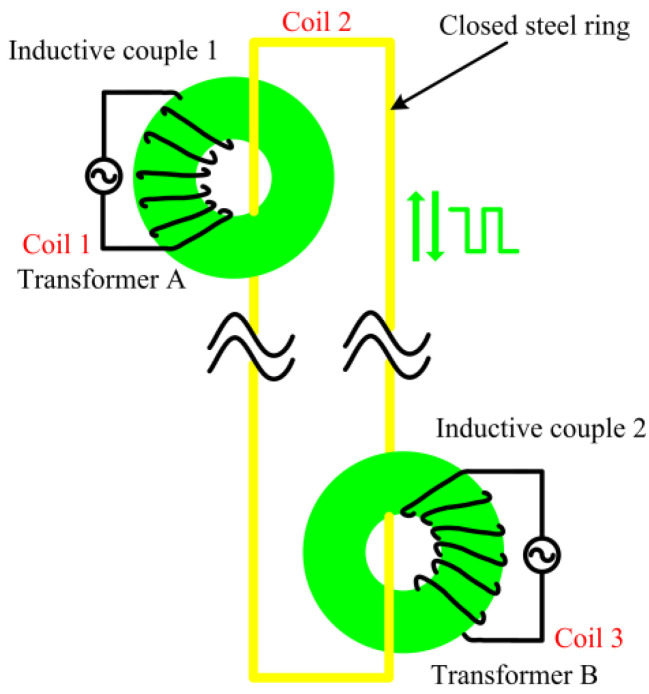
A schematic diagram of power feeding and data transmission.

A schematic diagram of the power feeding and data transmission is shown in Figure 6. The structure of the double transformer was devised to actualize the contactless power feeding and data transmission on the basis of the principle of the electromagnetic coupling. The CSR was not only the secondary coil of the transformer A but also the primary coil of the transformer B. According to the electromagnetic induction law [39,40,41,42], the input voltage *u_i_* of the transformer A is given by

(1) $$u_{i}=N_{1}\frac{d\varphi_{11}}{dt}=L_{1}\frac{di_{1}}{dt}$$

where *N*_1_ is the turns number of the coil 1, *L*_1_ is the self-inductance of the coil 1, *φ*_11_ is the main flux of the coupler, and *i*_1_ is the current following through the coil 1. Two coils cannot be fully coupled in actuality, and the coupling coefficient *k*_1_ is computed by [43,44,45,46]

(2) $$k_{1}=\frac{M}{\sqrt{L_{1}L_{2}}}$$

where *M* is the mutual inductance of the two coils, and *L*_2_ is the self-inductance of the coil 2. The relationship between the changes of the magnetic flux in the two-layer coils is as follows [39,40]:

(3) $$\frac{d\varphi_{12}}{dt}=k_{1}\frac{d\varphi_{12}}{dt}$$

where *φ*_12_ is the flux of the coil 2, and the voltage *u*_2_ of the coil 2 can be obtained according to the above equation.

(4) $$u_{2}=N_{2}\frac{d\varphi_{12}}{dt}=N_{2}k_{1}\frac{d\varphi_{11}}{dt}=k_{1}\frac{N_{2}}{N_{1}}u_{i}$$

where *N*_2_ is the turns number of the coil 2, and its value is 1 (single turn). Likewise, the voltage relationship of the transformer B can be obtained in the same way. Finally, the output voltage *u*_3_ is expressed by

(5) $$u_{3}=\frac{N_{3}}{N_{2}}u_{2}=k_{1}k_{2}\frac{N_{3}}{N_{1}}u_{i}$$

where *N*_3_ is the turns number of the coil 3, and *k*_2_ is the coupling coefficient of the transformer B.

**Figure 7:**
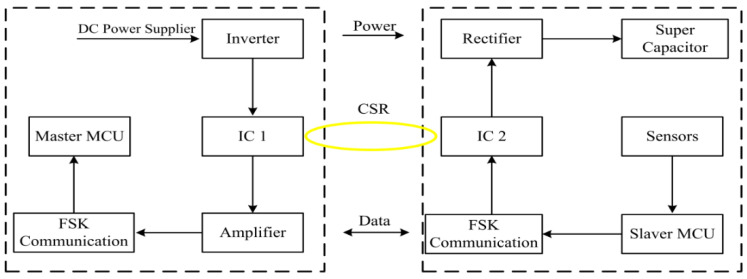
Block diagram of the system circuit.

The block diagram of the system circuit is shown in Figure 7. For the electromagnetic coupler, the principle of resonance is used to compensate the inductive reactance on the CSR. The supercapacitor is used as the energy temporary storage device of the sensor system, and the GMI is charged when it needs to work. After the power supply is stopped, the sensor uses the energy stored in the supercapacitor to collection and transmit data. At the same time, a simple and effective dual-power automatic switching circuit structure is proposed, which solves the contradiction between the direct power supply and the super capacitor power supply and becomes the core of the GMI high-efficiency power supply system. In the power transmission, the input signal of the primary coil (IC 1) is the obtained high frequency AC signal by a DC-AC inverter, and the power is transmitted to the CSR by electromagnetic coupling. The CRS is the secondary coil of the IC 1 and the primary coil of the IC 2 at the same time. Moreover, the electrical energy is transmitted to the sensing unit that provides the power for sensors.

## 4. Measuring Principle and Prediction Model of the Evaporation Duct

### 4.1. The Measuring Principle of the Evaporation Duct

The measuring principle of the evaporation duct can be divided into two categories. One is the direct measurement. According to the Debye’s theory [47,48], the modified atmospheric refractive index *M* that takes the Earth’s curvature into account can be expressed by

(6) $$M=\frac{77.6}{T}\times \left(p+\frac{4810e}{T}\right)+0.157z$$

where *p* (hPa) is the atmospheric pressure; *e* (hPa) is the water vapor pressure; *T* (K) is the atmospheric temperature; and *z* (m) is the altitude height above sea level. Moreover, many pairs of (*z*, *M*) at different heights are obtained, and nonlinear least-squares fitting is used to calculate *M* for each 0.1 m for all cases on the basis of a log-linear function given by

(7) $$M=f_{0}z-f_{1}\ln(z+0.001)+f_{2}$$

where the coefficients *f*_0_, *f*_1_, and *f*_2_ are calculated by using the least squares best fit. The constant 0.001 is applied to prevent the curve from blowing up at zero altitude. The other is the indirect measurement, and the *M* profile can be predicted on the basis of the Monin–Oboukhov similarity theory such as the Paulus–Jeske (PJ) model, the Musson-Genon–Gauthier–Bruth (MGB) model, the Naval Postgraduate School (NPS) model, and the Non-Iterative Air–Sea Flux (NAF) model. Owing to employing the advanced COARE 3.0 algorithm and stability correction function, the NAF model has plenty of advantages in predicting the *M* profile and is researched in this article.

### 4.2. The Non-Iterative Air–Sea Flux (NAF) Model

The models such as NPS, NAF, MGB, and PJ have been studied by researchers who carried out careful theoretical analyses and used the buoy data to validate and estimate these models [23,24]. Their results showed that the NPS model and NAF model are better than the MGB model and PJ model for estimating the height and the refractivity profile of the evaporation duct. Compared to the NPS model, the NAF model provides better computational efficiency and saves computational time for long-term and large-scale atmospheric duct simulation predictions. The evaporation detection system of the NAF model is simpler than log-linear fit. Thus, the NAF model is used to estimate the evaporation duct in this paper. At present, most of the flux calculation schemes of evaporation duct models require a large number of the operations or have a problem of low accuracy. In this paper, the EDH was not calculated by the whole transport method. Instead, according to the NAF model, the K theoretical flux observation method was introduced into the atmospheric refractive index equation, and the EDH was calculated by inputting the wind speed, temperature, relative humidity, pressure, and sea surface temperature at a certain height. The NAF model calculation scheme was formed by the regression analysis based on the calculation results of the PCB iteration scheme (Paulson70 parameterization scheme, CB05 flux scheme). In addition, the latest calculation scheme COARE3.0 was adopted to achieve the height correction of the evaporation duct under the condition of the large range roughness.

It is known that the atmospheric refractive index equation can be rewritten as [49,50]

(8) $$\frac{\partial N}{\partial z}=A+B\frac{\partial \theta }{\partial z}+C\frac{\partial q}{\partial z}$$

where *N* is the atmospheric refractive index, *q* is the specific humidity, and *θ* is the temperature. Moreover, the coefficients *A*, *B*, and *C* in the equation can be expressed as

(9) $$A=-0.01\times \rho g\left\{\frac{77.6}{T}+\frac{4810\times 77.6q}{T^{2}[\varepsilon +q(1-\varepsilon )]}\right\}+\frac{g[p-e(1-\varepsilon )]}{c_{pa}}\left\{\frac{77.6}{T^{2}}+\frac{2\times 4810\times 77.6q}{T^{3}[\varepsilon +q(1-\varepsilon )]}\right\}$$

(10) $$B=-{\left(\frac{p}{1000}\right)}^{R_{a}/c_{pa}}\left\{\frac{77.6p}{T^{2}}+\frac{2\times 4810\times 77.6qp}{T^{3}[\varepsilon +q(1-\varepsilon )]}\right\}$$

(11) $$C=\frac{4810\times 77.6p\varepsilon }{T^{2}{\left[\varepsilon +q(1-\varepsilon )\right]}^{2}}$$

where *T* is the air temperature, *p* is the atmospheric pressure, *ρ* is the air density, *e* is the water vapor pressure, *R_a_* is the dry air gas constant, *c_pa_* is the specific heat capacity at the constant pressure, and *ε* is the gas constant ratio.

In the NAF model [51], the turbulent flux *τ*, sensible heat flux *H_s_*, and water vapor flux *H_l_* are expressed by the following equations:

(12) $$\tau =\rho \bar{w^{\prime }u^{\prime }}=-\rho K_{m}\frac{\partial \bar{u}}{\partial z}=-\rho u_{*}^{2}$$

(13) $$H_{s}=\rho c_{p}\bar{w^{\prime }\theta^{\prime }}=-\rho c_{p}K_{h}\frac{\partial \bar{\theta }}{\partial z}=-\rho c_{p}u_{*}\theta_{*}$$

(14) $$H_{l}=\rho L_{e}\bar{w^{\prime }\theta^{\prime }}=-\rho L_{e}K_{q}\frac{\partial \bar{q}}{\partial z}=-\rho L_{e}u_{*}q_{*}$$

where *ρ* is the air density, *c_p_* is the specific heat capacity at the constant pressure, *u_∗_* is the characteristic scale of the wind speed, *θ_∗_* is the characteristic scale of the temperature, *L_e_* is the latent heat of the evaporation, and *q_∗_* is the characteristic scale of the specific humidity.

Substituting the first-order closed K theory and the near-surface turbulent flux expressions (13) and (14) into Equation (8), we can obtain

(15) $$\frac{\partial N}{\partial z}=A-BH_{s}\frac{1}{\rho c_{p}K_{h}}-CH_{l}\frac{1}{\rho L_{e}K_{q}}=A-H_{s}\frac{E}{K_{h}}-H_{l}\frac{F}{K_{q}}$$

(16) $$E=\frac{B}{\rho c_{p}}$$

(17) $$F=\frac{C}{\rho L_{e}}$$

where *K_h_* and *K_q_* are the turbulent heat exchange coefficient and turbulent water vapor exchange coefficient, respectively.

The height of the evaporation duct *Z_EDH_* usually satisfies

(18) $${\frac{\partial N}{\partial z}|}_{z=z_{EDH}}=-0.157$$

Combining the M-O similarity theory from Equations (15)–(17) and substituting it into Equation (18), at the height of the evaporation duct, we have

(19) $$A+0.157=\frac{E\varphi_{h}}{kz\sqrt{-\frac{\tau }{\rho }}}H_{s}+\frac{F\varphi_{q}}{kz\sqrt{-\frac{\tau }{\rho }}}H_{l}$$

where *k* is the Karman constant, and *z* is the input height parameter.

Substituting the stability correction function into the neutral condition (*z*/*L* = 0), the height of the evaporative duct is as follows:

(20) $$z_{EDH}=\frac{1}{k(A+0.157)}\sqrt{-\frac{\tau }{\rho }}(EH_{s}+FH_{l})$$

Under a stable condition (*z*/*L* > 0), the height of evaporation duct is as follows:

(21) $$z_{EDH}=\frac{(EH_{s}+FH_{l})[1+5.3\frac{\frac{z_{EDH}}{L}+{\frac{z_{EDH}}{L}}^{1.1}{\left(1+{\frac{z_{EDH}}{L}}^{1.1}\right)}^{-\frac{1}{11}}}{\frac{z_{EDH}}{L}+{\left(1+{\frac{z_{EDH}}{L}}^{1.1}\right)}^{\frac{1}{1.1}}}}{k(A+0.157)\sqrt{-\frac{\tau }{\rho }}}$$

Under an unstable condition (*z*/*L* < 0), the height of evaporation duct is as follows:

(22) $$z_{EDH}\sqrt{1-16\frac{z_{EDH}}{L}}=\frac{EH_{s}+FH_{l}}{k(A+0.157)\sqrt{-\frac{\tau }{\rho }}}$$

By choosing an appropriate iterative method, Equations (21) and (22) can be used to obtain the height of the evaporative duct under different atmospheric structuring states.

## 5. Test Experiment of the Magnetic Coupling System for Power Feeding and Data Transmission

**Figure 8:**
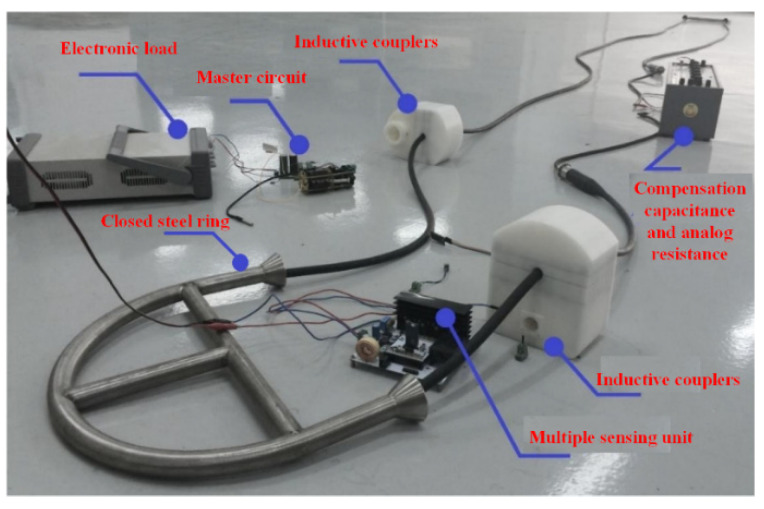
The power feeding and data transmission test experiment.

In order to verify whether the system can meet the performance requirements of the actual evaporation duct observation, the inductive couplers were used for power feeding and data transmission. The power feeding and data transmission test experiment is shown in Figure 8. The test system included the electronic load, closed steel ring, master circuit, inductive couplers, multiple sensing unit, compensation capacitance, and analog resistance. The preset indicators of the power feeding and data transmission system were as follows: the transmission power was 5~20 W; the power transmission efficiency reached 50%; the communication accuracy rate exceeded 99%.

**Figure 9:**
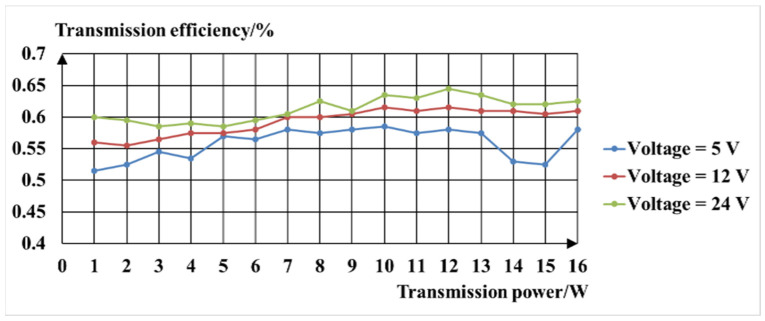
The relationship between transmission efficiency and transmission power.

The secondary coil of the inductive couplers converts AC energy into DC energy through a full bridge rectifier and capacitor filter, and then connects to the DC electronic load. The electronic load can simulate the load in the real environment. It has the working modes of the constant current, constant resistance, constant voltage, and constant power. It can set the load size by itself and automatically in order to stabilize at the set value; it is thus convenient for the testing of the system, since the sensing unit of the evaporation duct measurement system has an operating voltage range of 5~24 V and a power range of 1~15 W. During the experimental test, the measurement system provides square waves with amplitudes of ±24 V, ±12 V, and ±5 V. The electronic load of the simulation system was set to the constant power mode, and the range of the power was 1 W to 15 W. The voltage and current output were recorded by the stabilized voltage source every 1 W. The relationship between the transmission power and the transmission efficiency obtained by the experimental test is shown in Figure 9. The overall non-contact power transmission experiment showed that the overall power transmission efficiency of the system was about 57%, and the system transmission power reached 22.8 W, which essentially realizes the low-power transmission of non-contact power.

In order to verify the reliability of the communication, 1000 frames of data were sent by the sensing unit nodes to each other for 10 tests. There were three frames with the verification errors, and the correct reception rate was 99.7%. In addition, all error frames were automatically retransmitted. There was no check error after retransmission, and the communication continued smoothly. It can be seen that the communication was relatively stable and reliable. The communication error may have been caused by the residual magnetism accumulated in the magnetic core of the communication waveform of the previous frame, which affected the communication waveform of this frame, and the transmitted waveform was distorted, resulting in data error. The experimental test results showed that the system met the design requirements of the stable and reliable energy supply and data transmission, and also met the working requirements of the evaporation duct measurement system.

## 6. Results of the Evaporation Duct Measurement System

In order to measure the atmospheric refractive index profile, the atmospheric duct was calculated. The experiment was performed from 31 October 2021 to 5 November 2021 in the SCS. The data of the marine hydrological and meteorological sensors were obtained every three minutes in the experiment process. Each set of the experiment data contained five groups of data, which were mainly as follows: wind speed (WP), air temperature (AT), relative humidity (RH), air pressure (AP), and sea surface temperatures (SST). Considering the strong turbulence characteristics near the sea surface, the EDH needs to be determined by applying the Dybye theory to process and analyze the measured data.

### 6.1. A Sensitivity Study of Meteorological Data on EDH Based on the NAF Model

**Figure 10:**
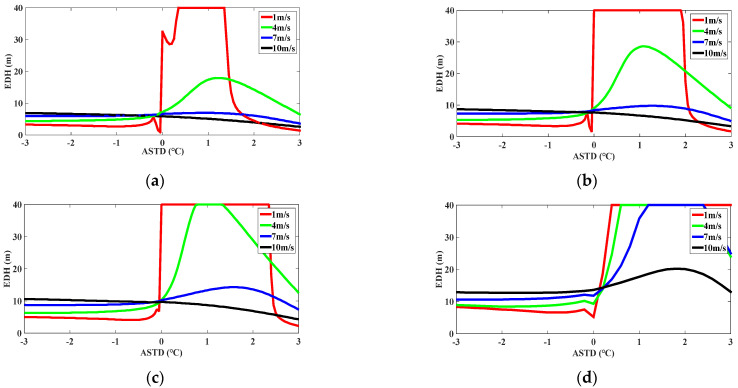
The predicted value of EDH according to the NAF model. The RH was fixed at 80%. The WS of 1 m/s, 4 m/s, 7 m/s, and 10 m/s are represented by the red solid line, green solid line, blue solid line, and black solid line, respectively. The SST was set at 15 °C, 20 °C, 25 °C, and 30 °C according to (**a**–**d**), respectively.

**Figure 11:**
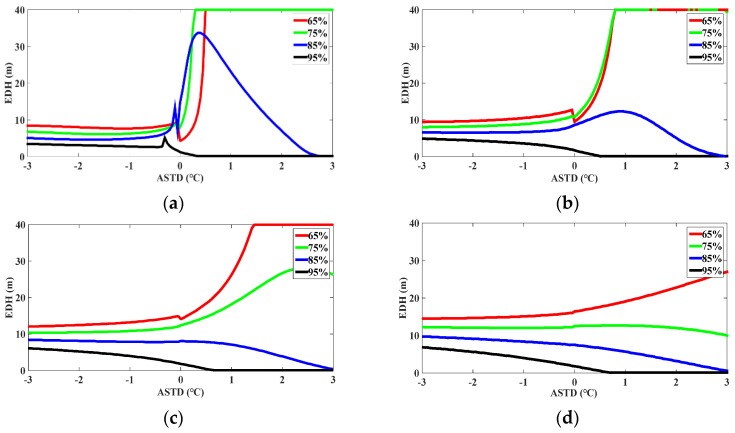
The predicted value of EDH according to the NAF model. The SST was fixed at 25 °C. The RH of 65%, 75%, 85%, and 95% are represented by the red solid line, green solid line, blue solid line, and black solid line, respectively. The WS was set to 1 m/s, 4 m/s, 7 m/s, and 10 m/s according to (**a**–**d**), respectively.

**Figure 12:**
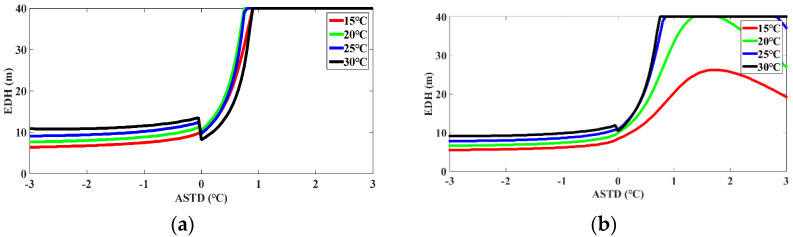

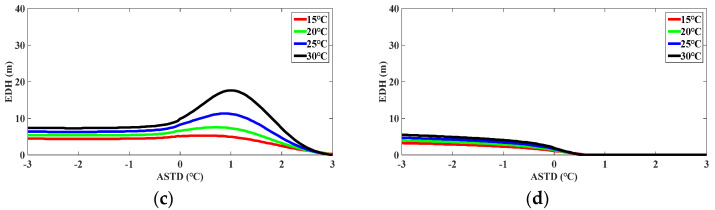
The predicted value of EDH according to the NAF model. The AT of 15 °C, 20 °C, 25 °C, and 30 °C are represented by the red solid line, green solid line, blue solid line, and black solid line, respectively. The WS was fixed at 5 m/s. The RH was set at 65%, 75%, 85%, and 95% according to (**a**–**d**), respectively.

Research shows that the EDH is mainly influenced by the ASTD, AT, RH, WP, and AP. The research on the adaptability of the model for predicting the evaporation duct propagation of the electromagnetic wave has an extremely important significance in radar communication and tactical guidance. To study the impact of the meteorological sensors on the EDH, the influence of the AT, RH, ASTD/and WP on the EDH is mainly analyzed by using the NAF model. The sensitivity of the meteorological sensors was simulated on the basis of the NAF model, as shown in Figure 10, Figure 11 and Figure 12. The horizontal axis is the ASTD, and the vertical axis is the EDH. The ASTD was less than or equal to or greater than zero, which is described as the instability, neutral, and stability conditions of the atmosphere, respectively. As the EDH is generally less than 40 m, it would be limited to 40 m, although it is greater than 40 m.

It can be seen from Figure 10 that under the unstable condition, with the ASTD increasing, the EDH essentially remained unchanged. When the ASTD remained unchanged, the EDH increased with the WS increasing; when the ASTD remained constant, the EDH increased slightly with the SST increasing. When transitioning from the unstable condition to the stable condition, the EDH rose rapidly and reached the maximum value as the ASTD continued to increase. When the WS decreased, the EDH increased faster. It shows that the evaporative duct was very sensitive to the change of the ASTD under the stable condition of the low WS. When the ASTD was constant, the EDH increased with the SST increasing under the stable condition.

As shown in Figure 11, under the unstable condition, the EDH decreased slowly with the RH increasing. Under the stable condition, when the RH was 95%, the EDH decreased rapidly to zero, and it can be interpreted as the trend of the rapid decline in the RH. When the RHs were 65% and 75% under the unstable condition in Figure 11a,b, respectively, the EDH rose rapidly to 40 m. When the RH was lower than 75% and the WS was 1 m/s or 4 m/s, the EDH increased rapidly to 40 m and remained unchanged at the EDH of 40 m with the ASTD increasing. When the RH was greater than 85%, the EDH also decreased rapidly with the ASTD increasing.

It can be seen from Figure 12a,b that when the RHs were 65% and 75%, respectively, and the ASTD was constant, and the EDH increased with the AT increasing under the unstable condition; when the AT was constant, the EDH was essentially stable under the stable condition, that is, the EDH was only somewhat affected by the ASTD when it was not stable. Under the stable condition, the EDH rapidly reached the maximum value with the ASTD increasing. In this case, the EDH was greatly affected by the SST. As shown in Figure 12c, when the RH was 85%, the EDH remained essentially unchanged under the unstable condition and the SST changed in the range of −3 °C to −0.8 °C; under the stable condition, the EDH first increased and then decreased with the ASTD increasing. When the ASTD was 1.2 °C, the EDH reached the maximum value. As shown in Figure 12d, when the RH was 95%, under the unstable condition, the EDH decreased slowly with the ASTD increasing; under the stable condition, the EDH decreased with the ASTD increasing. It decreased rapidly to zero, that is, there was no evaporative duct phenomenon under the stable condition.

According to the sensitivity study of the meteorological data in Figure 10, Figure 11 and Figure 12, under the unstable condition, when the ASTD was less than 0, the EDH was less affected by the ASTD. As the AT and SST rose, the WS increased higher, and as the RH decreased, the sea water evaporation became more favorable, a strong evaporative duct was formed, and the EDH was larger. However, under the stable condition, when the ASTD was greater than 0, the EDH was greatly affected by the ASTD. When the WS was relatively low, the RH was small and the ASTD was high, and it was easy to form a strong evaporation duct. In addition, it can also be seen that under the unstable condition, the sensitivity of the hydro meteorological sensor had little influence on the EDH. When the unstable condition was transited to the stable condition, the small mutation of the hydro meteorological parameters can cause the EDH to reach 40 m instantaneously, which would bring a great error to the prediction of the EDH. Thus, the meteorological data inaccuracies can bring up some huge errors under the stable condition. When the NAF model is used to predict the EDH, the prediction accuracy of the model can be improved by improving the sensitivity of the hydro meteorological sensor.

### 6.2. The Experiment of EDH Measurement in the South China Sea

In order to obtain the measured M values at each level of the tower platform, under the assumption of the static balance and ideal gas, the pressure at 8 m was converted to other levels by using the pressure height formula. Then, according to the Debye theory, the M values at different levels were able to be calculated by using Equation (6). Furthermore, the modified refractive index of the six layers can be fitted by using Equation (7) to perform the nonlinear least square fitting. The modified atmospheric refractive index profile and EDH were determined.

**Figure 13:**
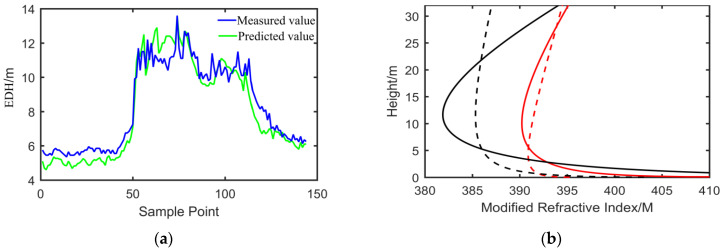
(**a**) The measured and predicted EDH from 0:00 to 23:59 in a day. The EDH was measured directly according to the Debye theory and also predicted on the basis of the NAF model with respect to the blue solid line and green solid line, separately. (**b**) Modified refractive index profile as the function of the height at two different moments with respect to the black line and red line, separately. The solid line represents the measured profile according the Debye theory, and the broken line expresses the predicted profile on the basis of the NAF model.

The EDH that varied from 4 to 14 m in a day is shown in Figure 13. The EDH first remained steady, then considerably mounted and levelled off, and finally decreased gradually; it can be interpreted as the evaporation of the sea water. In the morning, the air and sea water temperature were lower, which led to the weaker evaporation of the sea water and weaker EDH. The EDH increased rapidly with the air temperature and sea water temperature increasing. This phenomenon can be explained according to the aforementioned sensitivity study. Moreover, the EDH was small, and the occurrence probability of the evaporation duct was more than 95% in the SCS. The mean value of the EDH was 8.7 m, which was lower than the mean EDH of the SCS. The EDH trend of a day was in good agreement though the two methods. The modified refractive index profile as the function of the height is shown in Figure 13b. As shown in the black line, the EDH was identical, but there was a significant difference between the black solid line and black broken line. Because the black solid line was described by a log-linear function, it was obtained by Equation (7). However, the black broken line was obtained according to Equation (6). Sometimes, the shape of the modified refractive index profile was almost the same as that shown in the red line, but the value of the EDH showed an enormous difference, showing that the accuracy of the model requires further research.

## 7. Discussion

Since the NPS model has been used to evaluate the adaptability of the evaporation duct in the SCS [31,52], in order to verify the superiority of the NAF model in diagnosing the evaporation duct in the SCS, the sensitivity analysis results of the NAF model were effectively compared with the NPS model. For the variation of the WS, when the RH was 85% and the WS was less than 7 m/s, the EDH calculated by the NPS model and NAF model had a fast-rising trend with the ASTD changing. When the WS was greater than 7 m/s, the EDH calculated by the NPS model maintained a fast growth rate with the ASTD changing, while the NAF model showed a slightly decreasing trend with the ASTD changing, and the growth rate of the EDH tended to be flat. For the variation of the RH, when the SST was 25 °C, and the RH was less than 85%, the EDH calculated by the NPS model and NAF model had a fast-rising trend with the ASTD changing. When the RH was more than 85%, the decreasing rate of the EDH calculated by the NPS model and NAF model was obviously accelerated, and then tended to be flat. For the variation of the AT, when the WS was 5 m/s and the RH was 75%, the EDH calculated by the NPS model had a fast-rising trend with the ASTD changing, and the EDH calculated by the NAF model quickly rose to the EDH of 40 m and then tended to be flat. When the WS was 5 m/s and the RH was greater than 85%, the EDH calculated by the two models was kept consistent with the overall change trend, but the EDH calculated by the NPS model was slightly higher than that calculated by the NAF model.

According to the aforementioned analysis, the overall average EDH calculated by the NAF model was slightly lower than that calculated by the NPS model [31,52]. The sensitivity of the NAF model to the RH and WS under the unstable atmospheric stratification condition was consistent with that of the NPS model; under the stable atmospheric stratification condition, the NAF model had an obvious response to the variation of the ASTD, RH, and WS. The diagnosis effect of the NAF model was better than that of the NPS model, mainly because the NAF model adopts a more optimized and reasonable non-iterative flux parameterization scheme, and its calculation results are close to the Babin model and the NPS model using the latest algorithm COARE3.0 [31,52]. It can be seen that the computational stability of the NAF model was better, the probability of extreme values was lower, and the diagnostic results were relatively reliable. In general, the simulation effect of the NAF model was more stable when different meteorological elements changed.

## 8. Conclusions

A novel system for the measurement of the evaporation duct on the basis of the magnetic coupling technology for the power feeding and data transmission was proposed in this paper. No direct connection between the power source and load was realized. The stability of the system, especially in high humidity and high salt fog conditions, can be improved. The magnetic coupling system was designed by some simulations and experiments in detail. The sensitivity of the NAF model was analyzed, and the EDH was compared with the measured value on the basis of the experiment data in the SCS. The EDH was detected by not only the direct measurement but also the indirect prediction based on the NAF model. The results show that the system for measuring the evaporation duct using the magnetic coupling technology for the power feeding and data transmission was feasible in practice. The overall efficiency of the power transmission reached 57%, and the transmission power was 22.8 W, which realized the low-power transmission of GMI non-contact power. The communication of the whole system was stable and reliable, and the correct rate of data communication for 1000 frames in the experiment reached 99.7%. On the basis of the analysis of the experimental data of the SCS, it was found that the prediction trend of the EDH by the NAF model was essentially consistent with the measured value. The mean value of the EDH was 8.7 m, which was lower than the mean EDH of the SCS. The EDH calculated by the NAF model in the unstable air–sea stratification state was slightly lower than that calculated by the NPS model. The diagnosis of the EDH by the NAF model was similar to that of the NPS model, but the calculation stability of the NAF model was better.

Although a novel gradient observation system was proposed in this paper, the atmospheric refractive rate profile was obtained by using the NAF model. It verified the fact that the model can perform an effective evaporative duct diagnosis and prediction under the unstable air–sea stratification in the SCS. However, the electromagnetic coupler with a toroidal core was only used as the non-contact power transmission structure of the GMI. In the future, the non-contact power transmission efficiency can be further improved by studying the influence of other special-shaped electromagnetic coupler structures on the system transmission efficiency.

The prediction of the EDH by a single NAF model under the neutral or the unstable state would bring about large errors to the experimental results. In view of this problem, in the follow-up study of the NAF model, a variety of model fusion methods (PJ, Babin, NPS, and other models) can be used to perform the data fusion joint prediction according to different weights. It reduces the large error caused by a single NAF model to the height diagnosis results of the evaporation duct. When the linear relationship is selected under the stable condition of the NAF model, it would lead to unreasonable EDH results. This unreasonable phenomenon can be improved by developing some new universal functions under stable conditions. Moreover, due to the limited experimental data at sea and the inconsistent horizontal distribution characteristics of the different sea areas, a large number of empirical formulas and assumptions were used in the prediction of the NAF model, and the accuracy of the NAF model was not the same. To apply the NAF model to the actual sea environment, a long-term comparative analysis test needs to be carried out. In later research, the capacitive refractometer can be considered to obtain the atmospheric refractive rate profile by measuring the resonant frequency of the resonator. Similarly, the lidar system can also be used to obtain atmospheric water vapor pressure parameters by emitting electromagnetic waves in different bands, and then the atmospheric refractive rate profile is obtained. The accuracy of the atmospheric refractive rate profile of the NAF model is further verified.

## Figures and Tables

**Table 1:** Parameters of all sensors.

Sensors	Measuring Range	Measuring Accuracy
Wind speed (m/s)	0.5~90	±0.2
Relative humidity (%)	0~100	±1 (0~90), ±2 (90~100)
Air temperature (°C)	−30~50	±0.2
Air pressure (hPa)	600~1100	±1.5
Sea surface temperature (°C)	−30~50	±0.5

## Data Availability

Not applicable.
